# Regionally representative hair mercury levels in Canadian First Nations adults living on reserves

**DOI:** 10.17269/s41997-021-00508-5

**Published:** 2021-06-28

**Authors:** Constantine Tikhonov, Harold Schwartz, Lesya Marushka, Hing Man Chan, Malek Batal, Tonio Sadik, Amy Ing, Karen Fediuk

**Affiliations:** 1 First Nations and Inuit Health Branch, Indigenous Services Canada, Ottawa, ON, Canada; 2 Department of Biology, University of Ottawa, 30 Marie Curie, Ottawa, ON K1N 6N5 Canada; 3 Département de Nutrition, Faculté de Médecine, Université de Montréal, Pavillon Liliane de Stewart, C.P. 6128, succ. Centre-Ville, Montréal, QC H3T 1A8 Canada; 4 Centre de recherche en santé publique de l’Université de Montréal et du CIUSS du Centre-sud-de-l’Île-de-Montréal (CReSP), 7101 Avenue du Parc, Montréal, QC, H3N 1X7 Canada; 5 Assembly of First Nations, 55 Metcalfe Street, Suite 1600, Ottawa, ON K1P 6L5 Canada

**Keywords:** Mercury, Indigenous, First Nations, Biomonitoring, Hair, Mercure, Autochtones, Premières Nations, biosurveillance, cheveux

## Abstract

**Objective:**

The primary objective of this participatory study was to assess the current body burden of mercury among First Nations adults.

**Methods:**

The First Nations Food, Nutrition and Environment Study (2008–2018) collected regionally representative data from First Nations adults living on reserves south of the 60^th^ parallel. Mercury was analyzed in hair as a preferred biomarker for prolonged exposure. Hair samples, a 5 mm bundle cut from the occipital region, were collected from the participants who gave consent and measured for total mercury concentrations using cold vapor atomic fluorescence spectrophotometry.

**Results:**

In total, 3404 First Nations adults living in 92 communities provided hair samples. This represents 52.5% of the respondents to the household surveys. The mean hair mercury concentrations were 0.56 μg/g among all participants and 0.34 μg/g among women of childbearing age (WCBA). There were 64 exceedances of Health Canada’s mercury biomonitoring guidelines (44 WCBA, 8 women aged 51+ years, 3 men aged 19–50 years, and 9 men aged 51+ years).

**Conclusion:**

Current mercury exposure no longer presents a significant clinical health risk in most of the First Nations population south of the 60^th^ parallel across Canada. However, mercury exposure continues to be an ongoing environmental public health concern that requires continued monitoring and assessment. Women of childbearing age (19–50 years) and older individuals living in northern ecozones and Quebec have higher mercury exposures, often exceeding Health Canada’s guidelines. Careful risk communication and risk management programs need to focus on northern ecozones and Quebec.

## Résumé

**Objectif:**

L’objectif principal de cette étude participative était d’évaluer la charge corporelle actuelle de mercure chez les adultes des Premières Nations.

**Méthodes:**

L’Étude sur l’alimentation, la nutrition et l’environnement chez les Premières Nations (2008–2018) a permis de recueillir des données régionales représentatives auprès d’adultes des Premières Nations vivant dans des réserves au sud du 60^e^ parallèle. Le mercure a été analysé dans les cheveux; il s’agit d’un biomarqueur de choix en cas d’exposition prolongée. Des échantillons de cheveux, soit une mèche de 5 mm coupée de la région occipitale, ont été recueillis auprès des participants qui ont donné leur consentement, et les concentrations totales de mercure ont été mesurées par spectrophotométrie de fluorescence atomique à vapeur froide.

**Résultats:**

Au total, 3 404 adultes des Premières Nations vivant dans 92 communautés ont fourni des échantillons de cheveux, ce qui représente 52,5 % des répondants aux enquêtes auprès des ménages. Les concentrations moyennes de mercure dans les cheveux étaient de 0,56 μg/g pour l’ensemble des participants et de 0,34 μg/g pour les femmes en âge de procréer. Il y a eu 64 dépassements des valeurs de biosurveillance du mercure recommandées par Santé Canada (44 femmes en âge de procréer, 8 femmes de 51 ans et plus, 3 hommes de 19 à 50 ans et 9 hommes de 51 ans et plus).

**Conclusion:**

L’exposition actuelle au mercure ne présente plus un risque important pour la santé clinique de la plupart des populations des Premières Nations au sud du 60^e^ parallèle au Canada. Toutefois, l’exposition au mercure demeure une préoccupation de santé publique environnementale qui doit faire l’objet d’une surveillance et d’une évaluation continues. Les femmes en âge de procréer (de 19 à 50 ans) et les personnes âgées vivant dans les écozones nordiques ou au Québec sont plus exposées au mercure, exposition qui dépasse souvent les valeurs recommandées par Santé Canada. Des programmes de communication et de gestion des risques rigoureux doivent être axés sur les écozones nordiques et le Québec.

## Introduction

Mercury (Hg) is widely spread and persistent in the environment (AMAP/UN Environment, 2019). Hg is emitted by natural sources such as forest fires, volcanoes, and geologic deposits. However, anthropogenic sources, including coal-burning, metals smelting, gold and silver mining, and chlor-alkali production using mercury or mercury compounds, can emit equal amounts or even more Hg in the environment (UN Environment, 2013, 2019). Mercury also enters the environment from incinerators and from areas flooded by dams, and through the disposal of old products containing mercury. Anthropogenic emissions, in gaseous elemental form, are relatively stable in the atmosphere (Kim et al., 2016; Ma et al., 2019). Mercury can be transported by air currents over very long distances and then deposited on the landscape and in the ocean, where it is further transformed into different chemical forms (ECCC, 2016; Schroeder & Munthe, 1998).

Methylmercury is one of the most toxic forms of mercury, affecting the central nervous system, particularly in developing fetuses and young children. It also disturbs immune function, alters genetic and enzyme systems, and is linked to increased risk of cardiovascular diseases (Bjørklund et al., 2017; Ha et al., 2017). The primary source of ongoing chronic exposure to methylmercury in human populations is through the consumption of fish and sea mammals (Clarkson et al., 2003). Generally, predatory fish (such as mackerel, walleye, and pike) tend to have higher concentrations of methylmercury due to bioaccumulation and biomagnification (Health Canada, 2008; Driscoll et al., 2013). Levels of mercury in fish vary by species, length, sex, and ecozone due to associated physical and chemical variations in the environment that influence Hg bioaccumulation in aquatic food webs (Burgess et al., 2016). An ecozone is a large geographical region characterized by a distinct biodiversity of flora and fauna (www.ecozones.ca).

Indigenous people, including First Nations, are particularly vulnerable to higher mercury exposure due to their reliance on traditional foods, including fish (Kuhnlein & Chan, 2000). Indeed, very high levels of mercury exposure have been well documented previously among the First Nations and Inuit populations in Canada (Wheatley & Paradis, 1995; Donaldson et al., 2010; Curren et al., 2014). It was realized by 1970 that methylmercury contamination of river systems results from the pollution of the systems with inorganic mercury, which is converted to the toxic methylated form by natural bacterial processes in sediments and the water column of the ocean and large lakes, but not in the water of most freshwater systems. The complexity of mercury cycling in aquatic systems is a subject of ongoing research (Health Canada, 1979; AMAP/UN Environment, 2019). In the 1960s and early 1970s, 10,000 kg of inorganic mercury was released into the English-Wabigoon River system from a chlor-alkali plant located near Dryden in Ontario (Kinghorn et al., 2007). In 1971, 65 residents of two First Nations communities in northwestern Ontario (Asubpeeschoseewagong Netum Anishinabek and Wabaseemoong Independent Nations, also known as Grassy Narrows and Whitedog First Nations) were examined by a team from the Ontario Ministry of Health. Nearly 50% of the sampled individuals had levels over 100 μg/L in blood (Health Canada, 1979). The Cree populations of James Bay, in Quebec, have also been exposed to high levels of methylmercury through the consumption of contaminated fish from natural lakes and hydroelectric reservoirs (Dumont et al., 1998). A study of mercury exposure involving 49 Cree and Algonquin participants in northwestern Quebec (Barbeau et al., 1976, cited from Schoen & Robinson, 2005) reported that at least six and possibly 25 of the 49 individuals examined showed “definitive objective signs of neurological impairment”, associated with mercury toxicity (Health Canada, 1979).

During the early 1970s, the Medical Services Branch of Health Canada (now the First Nations and Inuit Health Branch of Indigenous Services Canada, hereafter FNIHB) became involved in the initial investigations of blood and hair mercury levels among First Nations residents in Ontario and Quebec (Health Canada, 1979). In 1973, a Task Force on Organic Mercury in the Environment was established by Health Canada “in order to respond to the problem of high and unusual mercury levels in relation to the health and well-being of residents of Grassy Narrows and Whitedog, Ontario” (Legrand et al., 2010). In 1975, recognizing multiple potential sources of mercury exposure in the environment, FNIHB expanded the systematic mercury biomonitoring program among First Nations and Inuit, making it national in scope. This *Methylmercury in Canada* Program originated as a public health surveillance, aiming to assess and find means to mitigate the extent of mercury exposure and the associated health risk among Indigenous Peoples. Between 1970 and 1992, a total of 72,556 hair and blood tests for mercury among 40,634 individuals were carried out in 529 communities across Canada (Health Canada, 1999). To identify “at risk” individuals and provide appropriate preventive action, FNIHB/Health Canada established a set of biomonitoring guidelines (Health Canada, 1979). The levels of mercury in blood below 20 μg/L were considered to be in the “normal acceptable range”; from 20 to 100 μg/L, “increasing risk”; and above 100 μg/L, “at risk”. The corresponding hair guidance levels were 6 μg/g and 30 μg/g (Health Canada, 1999). The guidance values were based on the recommendations of the 1971 Swedish Expert Group (SEG) report, which concluded that the lowest blood concentration associated with adverse clinical effects was approximately 200 μg/L. This analysis was based on the findings from investigations of large outbreaks of organic mercury poisoning—in Japan in the 1950s–1960s and Iraq in the 1970s. The expert group recommended applying a safety factor of 10 to derive “safe” levels in human populations (SEG, 1971; cited from Health Canada, 1979). However, a multitude of research conducted over the last three decades suggests that mercury exposure is associated with detrimental health effects due to prenatal exposure (e.g., low birth weight, subtle delays in neurobehavioural development, impacts on working memory in children) at levels below the Health Canada biomonitoring guidelines for the general population (Ha et al., 2017; Kim et al., 2016; Basu et al., 2018). While the continued focus on early stages of life remained of primary importance, some low levels of mercury exposures were reported to be associated with a higher risk of developing neurological and cardiovascular disease, as well as some reproductive outcomes (Kim et al., 2016; Chan, 2019; Thomas et al., 2015).

In 2010, Health Canada adopted additional biomonitoring guidelines, applicable specifically to women of childbearing age (WCBA) and children. The new proposed level of concern was set at 2 μg/g in hair (8 μg/L in blood, based on the conversion factor of 250) (Legrand et al., 2010). The new blood guidance for mercury harmonized the WCBA biomonitoring with the provisional tolerable daily intake (pTDI) guidance developed by Health Canada for pregnant women, women of reproductive age, and infants, set at 0.2 μg/kg bw/day (Feeley & Lo, 1998).

The biomonitoring component of the First Nations Food, Nutrition and Environment Study (FNFNES) relied on scalp hair sampling for mercury analysis. Blood and hair mercury concentrations are conventional biomarkers for methylmercury exposure. The blood mercury concentration is directly proportionate to the mercury concentration in new hair, although it has been reported that the maximum hair segment mercury concentration appears 20 days after the maximum concentration in the blood (NRC, 2000; Hislop et al., 1983). The ratio of hair mercury to blood mercury varies among individuals, but the average is about 250:1 (JECFA/WHO, 2004; Kales & Christiani, 2005). The average rate of scalp hair growth is estimated to be about 1 cm/month (Grandjean et al., 2002). Hair is a preferred biomarker for estimating mercury body burden due to its relatively non-invasive collection and the ability to estimate month-to-month mercury levels in longer hair samples.

The primary objective of this study was to present and describe the first regionally representative picture of the mercury body burden among First Nations people living on reserves in Canada, based on hair analysis. A comparison with historical levels (1970–1996) and with chronic low-level mercury body burden in the general population in Canada is also provided. The risk of mercury exposure was assessed by comparing the hair mercury concentrations in FNFNES participants with Health Canada guidelines.

## Methodology

### Study design

**Fig. 1:**
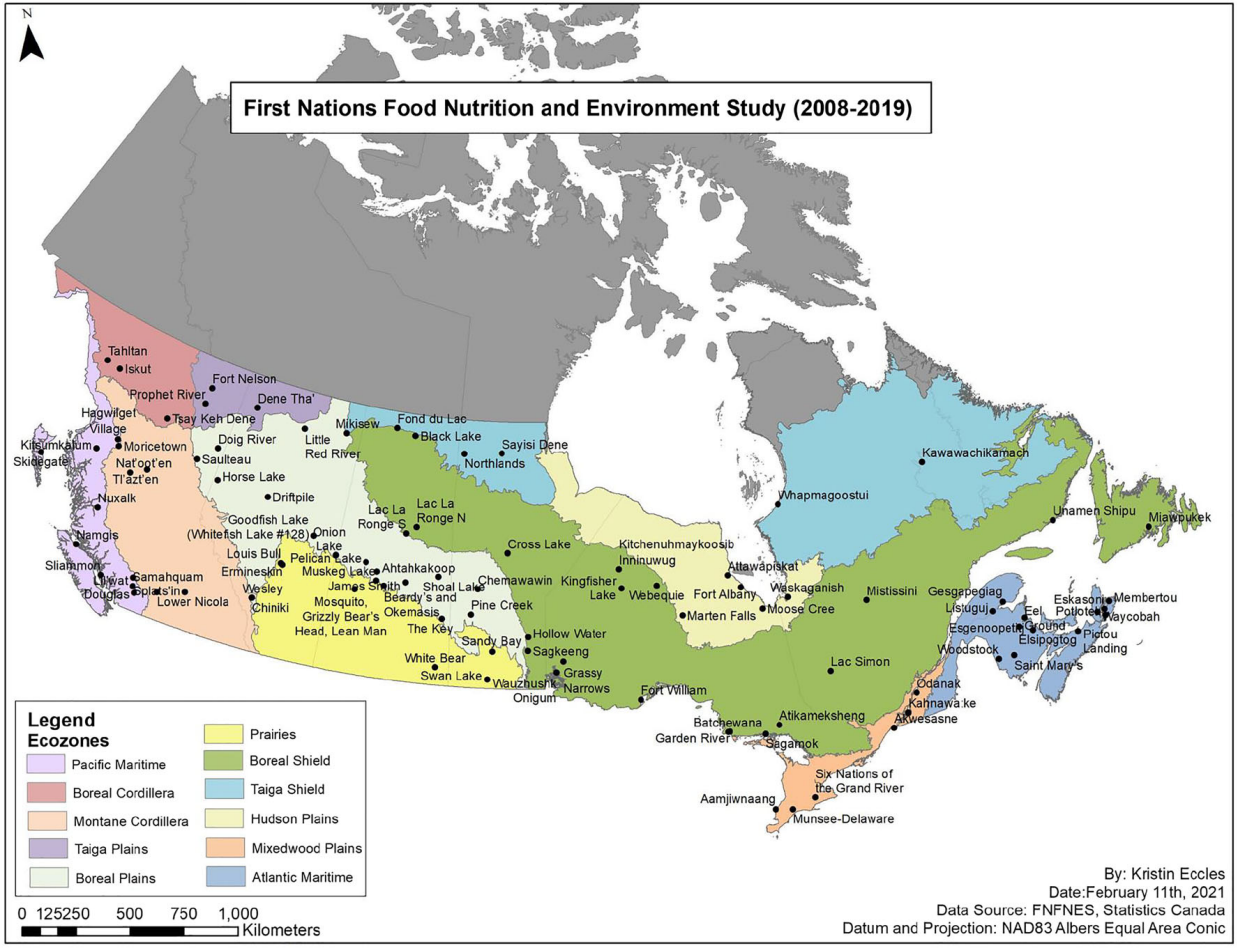
Map of First Nations communities participating in the FNFNES

The FNFNES was implemented in the eight Assembly of First Nations (AFN) regions over a 10-year period (2008–2018) and is regionally representative of all First Nations adults living on reserves south of the 60^th^ parallel. A total of 92 First Nations completed the five general study components of FNFNES (Fig. 1). It should be noted that members from one First Nation occupied reserves in two ecozones; therefore, a decision was made to split the First Nation into two sites by an ecozone boundary. Consequently, many tables in this special issue of CJPH describe a total of 93 First Nations at the AFN region and ecozone levels (e.g., Table 1).

**Table 1:** Sample characteristics by AFN regions (Atlantic Region includes New Brunswick, Nova Scotia, and Prince Edward Island): number of communities and hair mercury sampling participants

	Total	British Columbia	Manitoba	Ontario	Alberta	Atlantic	Saskatchewan	Quebec & Labrador
Year(s) of data collection	2008–2016	2008–2009	2010	2011–2012	2013	2014	2015	2016
First Nations, *n*	93	21	9	18	10	11	14	10
FNFNES participants, *n*	6487	1103	706	1429	609	1025	1042	573
Hair Hg sample participants, *n*	3404	487	236	744	369	632	555	381
Participation rate, %	52.5	44.2	33.4	52.1	60.6	61.7	53.3	66.5
Males, *n*	969	141	38	236	121	188	157	88
Females, *n*	2435	346	198	508	248	444	398	293
WCBA (19–50 years), *n*	1607	246	138	302	176	296	269	180
N (%) ≥ LOD^*§#^	2588 (76.0)	459 (94.3)	169 (71.6)	645 (86.7)	238 (64.5)	422 (66.8)	348 (62.7)	307 (80.6)
Total population, Hg range, μg/g	<LOD–23.5	<LOD–4.6	<LOD–8.9	<LOD–13.5	<LOD–7.2	<LOD–3.7	<LOD–11.6	<LOD–23.5
WCBA, Hg range, μg/g	<LOD–8.9	<LOD–4.6	<LOD–8.9	<LOD–5.3	<LOD–2.7	<LOD–1.4	<LOD–5.2	<LOD–6.0

*WCBA*, women of childbearing age; *LOD*, limit of detection

^*^LOD is defined as Hg detected in at least one of three 1 cm hair samples

^§^LOD were as follows: British Columbia—0.06 μg/g; Manitoba—0.06 μg/g; Ontario—0.06 and 0.07 μg/g; Alberta, Atlantic, Saskatchewan, and Quebec & Labrador—0.07 μg/g

^#^Unweighted estimates

The details of the community-based participatory study design and sampling of First Nations communities, as well as its limitations, are described in Chan et al. (2019b, 2021b) in this CJPH special issue. Comprehensive region-by-region information on the study implementation is also available online (http://www.fnfnes.ca).

### Ethics

Individual participation in the project was voluntary and based on informed written consent after an oral and written explanation of each project component. This survey was conducted following the “Tri-Council Policy Statement: Ethical Conduct for Research Involving Humans” and, in particular, Chapter 9, concerning research involving the First Nations, Inuit, and Métis Peoples of Canada. The Ethical Review Boards at the University of Northern British Columbia, the University of Ottawa, the Université de Montréal, and Health Canada approved the study. The FNFNES respected the First Nations principles of Ownership, Control, Access, and Possession (OCAP®) of data (Schnarch, 2004).

### Hair sampling for mercury analysis

Participation in hair sampling was voluntary. Hair samples were collected during household interviews in the fall of each study year (from 2008 to 2016). In essence, a 5 mm bundle of hair was isolated and cut from the back of the head, ensuring a minimal and most often unnoticeable effect on participants’ aesthetics. The hair bundle (full length, as cut from the scalp) was placed in a polyethylene bag and fastened to the bag with staples near the scalp end of the hair bundle. For participants with short hair, a short hair sampling procedure was followed. For this procedure, approximately 10 mg of hair was trimmed from the base of the neck onto a piece of paper. The paper was then folded, stapled, and placed in a polyethylene bag (Chan et al., 2019a).

### Laboratory analysis of hair samples

All hair samples, accompanied by a duly filled chain of custody form, were sent by the national study coordinator to the FNIHB co-investigator, who entered all data associated with the hair samples (participant identification number, age, and sex) into a spreadsheet. The hair samples were then sent to the First Nations and Inuit Health Branch (FNIHB) Laboratory in Ottawa, Ontario (for British Columbia, Manitoba, and Ontario regions) or to the Health Canada Quebec Region Laboratory in Longueuil, Quebec (for Alberta, Atlantic, Saskatchewan, and Quebec/Labrador regions) for analysis. The FNIHB Laboratory was accredited by the Canadian Association for Laboratory Accreditation (CALA) to the standard ISO/IEC 17025 and participated in the International Hair Mercury Inter-Laboratory Comparison Program. The Health Canada Quebec Region Laboratory was accredited by the Standards Council of Canada (SCC) to ISO/IEC 17025. No information that could be used to identify the participant was included in the package sent to FNIHB.

In the laboratory, each hair bundle was cut into 1 cm segments, starting from the scalp end. Three segments were analyzed to provide an assessment for Hg exposure in approximately the last 3 months. For short hair samples (less than 1 cm), the level of mercury is only available for less than 1 month. Total mercury and inorganic mercury (segments with levels greater than 1.0 μg/g) in the hair were analyzed.

Segmented hair samples were chemically treated to release ionic mercury species, which were further selectively reduced to elemental mercury. The latter was concentrated as its amalgam using gold traps. The mercury was then thermally desorbed from the gold traps into the argon gas stream, and the concentration of mercury vapours was measured with a UV-detector at 254 nm wavelength using a cold vapor atomic fluorescence spectrophotometer (CVAFS). Selective reduction of the ionic mercury species allowed the measurement of total or inorganic mercury. There were changes in the limit of detection (LOD) for total mercury across the years of the study from 0.06 μg/g (in BC) to 0.06 and 0.07μg/g in other regions (Table 1). The LOD for inorganic mercury in hair was 0.02 μg/g. For statistical analysis, the measurement results below the level of detection were substituted by a value equal to LOD/2, which was considered to be sufficiently accurate for this application (Kushner, 1976). For QA/QC purposes, duplicate analyses were run on 10% of the samples in both participating laboratories. Any unused hair left from the original bundle was replaced in the polyethylene bag and, together with unused segments, returned to participants at the end of each study year.

### Data analyses

Statistical analyses were performed using SAS Enterprise Guide, Version 5.1 (The SAS Institute Inc., Cary, NC, USA) and STATA statistical software, 14.2 (StataCorp, College Station, Texas, USA). Descriptive statistics included the calculation of arithmetic and geometric means with 95% confidence interval (CI), and 95th percentile with 95% CI for different age and sex groups. All estimates were weighted to obtain representative data at the regional level. The weighting variables were adjusted for the population growth from 2008 to 2017.

#### Data comparisons

The study results were compared with historical results in the First Nations population, based on the *Methylmercury in Canada* Program (Health Canada, 1979, 1999). There was a methodological difference in approaches used in the collection of biomonitoring data for the historical surveys and FNFNES. The key difference was in the purpose of the biomonitoring investigation undertaken in 1970–1996, which was to conduct systematic public health surveillance of mercury exposure among high consumers of fish in First Nations communities. The sampling was not random, but based on volunteers in First Nations communities, who had self-identified as fishing guides and/or high consumers of fish (Wheatley & Paradis, 1995), whereas participation in the FNFNES was based on systematic random sampling and testing occurred at one time point during the fall with the aim of estimating mercury body burden at the regional level among First Nations adults. Therefore, the prevalence of exceedances based on the historical biomonitoring data were compared with both the FNFNES total population and the study participants aged 51+ who tend to consume more fish and other traditional foods. The methodological difference suggests that we cannot draw direct comparisons between historical and current results. However, keeping this limitation in mind, it is essential to examine key differences in levels of mercury exposure determined on the basis of these large samples of the First Nations population.

The FNFNES mercury results were also compared with the general Canadian population, based on the results of the Canadian Health Measures Survey (CHMS) from cycle 1 (2007/2008) to cycle 4 (2014/2015). The CHMS is an ongoing national direct health measures survey conducted by Statistics Canada, Health Canada and the Public Health Agency of Canada, which was launched in 2007 to collect nationally representative health and wellness data and biological samples of Canadians (Health Canada, 2019). Due to the difference in the age groups between the two studies, custom data tables were developed by Health Canada’s National Biomonitoring Section (HECSB) to present data for total mercury measurements in whole blood for the Canadian population aged 19–79 years. Methodological notes on the data specified that there were changes in the LOD across CHMS cycles, which had an impact on the %<LOD as well as the data. It was also noted that some data were flagged for high variability. Estimates with coefficients of variation greater than 16.6 were shown with a warning (“E”), while those with coefficients of variation greater than 33.3 were suppressed (“F”) (T. Pollock and A. St-Amand. National Biomonitoring Section, Healthy Environments and Consumer Safety Branch, Health Canada; personal communication, 2019).

Finally, mercury results were compared with Health Canada’s guidelines of 2 μg/g in hair (8 μg/L in blood) for WCBA and children from birth to 18 years. The guideline for adult males (>18 years) and women aged 51+ is higher, at 6 μg/g in hair (or 20 μg/L in blood). There is also an “action level” of mercury exposure set at 30 μg/g in hair or 100 μg/L in blood, which applies to the general population and requires medical consultation and potential intervention (Legrand et al., 2010). It is important to note that the older guidelines (20–100 μg/L in blood measurements) are provided with legacy hair equivalence values (6–30 μg/g), based on the conversion factor of 300 (Health Canada, 1979). The more recent set of guidelines, applicable to WCBA, use the conversion factor of 250, which is the current international consensus value (JECFA/WHO, 2004).

## Results

In total, 3404 First Nations adults (2432 women and 972 men) agreed to have their hair sampled and tested for mercury (Table 1). This represents 52.5% of the respondents to the household surveys. At the regional level, the participation rates ranged from 33.4% to 66.5%. Mercury was detected in 76% of the sampled individuals. The proportions of respondents with hair mercury concentration below the LOD varied between age and sex categories and between regions (from 5.7% in BC to 37.3% in SK) (Table 1). Mercury component estimation weights were calculated for each region based on the data on hair mercury samples. All estimates on hair mercury concentrations were weighted unless otherwise stated.

The majority of respondents to the mercury component were females (71.4%), and a higher proportion of females (66.1%) were of childbearing age, i.e., 19–50 years. Among men, the lowest participation rate (16.1%) was observed in Manitoba. Sample characteristics by region are presented in Table 1.

The mean concentration of total mercury in hair among First Nations adults was 0.56 μg/g, with a 95% confidence interval between 0.43 and 0.69 μg/g. Mean mercury concentrations varied between regions (Table 2). The highest arithmetic means of hair mercury concentration were observed among First Nations living in Quebec (1.39 μg/g), British Columbia (0.58 μg/g), Manitoba (0.42 μg/g), and Ontario (0.40 μg/g) (while the geometric means for the corresponding regions were 0.39 μg/g, 0.37 μg/g, 0.15 μg/g, and 0.19 μg/g, respectively). First Nations living in the Atlantic region had the lowest level of hair mercury with the arithmetic mean at 0.18 μg/g and the geometric mean at 0.10 μg/g. Among WCBA, the highest average concentrations of hair mercury were reported in Quebec (0.74 μg/g), British Columbia (0.42 μg/g), and Ontario (0.30 μg/g). Overall, men had higher concentrations of mercury in hair than women did (*p* = 0.0112). Also, mercury exposure increased with age among all participants (*p* < 0.001), which was statistically significant over age groups for both men (*p* < 0.01) and women (*p* < 0.001). The same tendency is seen in the analysis by region.

**Table 2:**
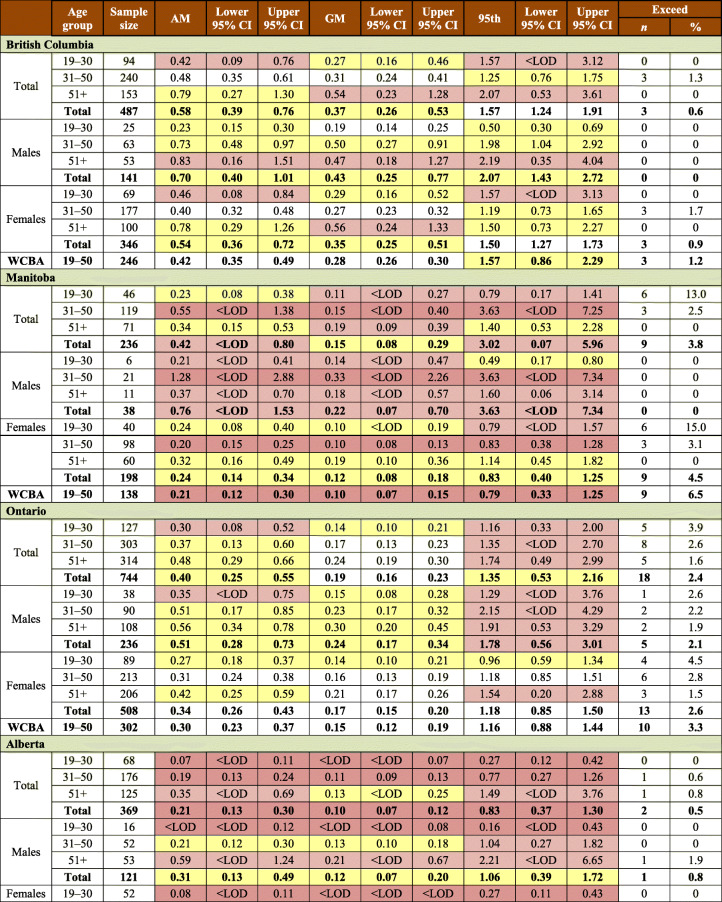

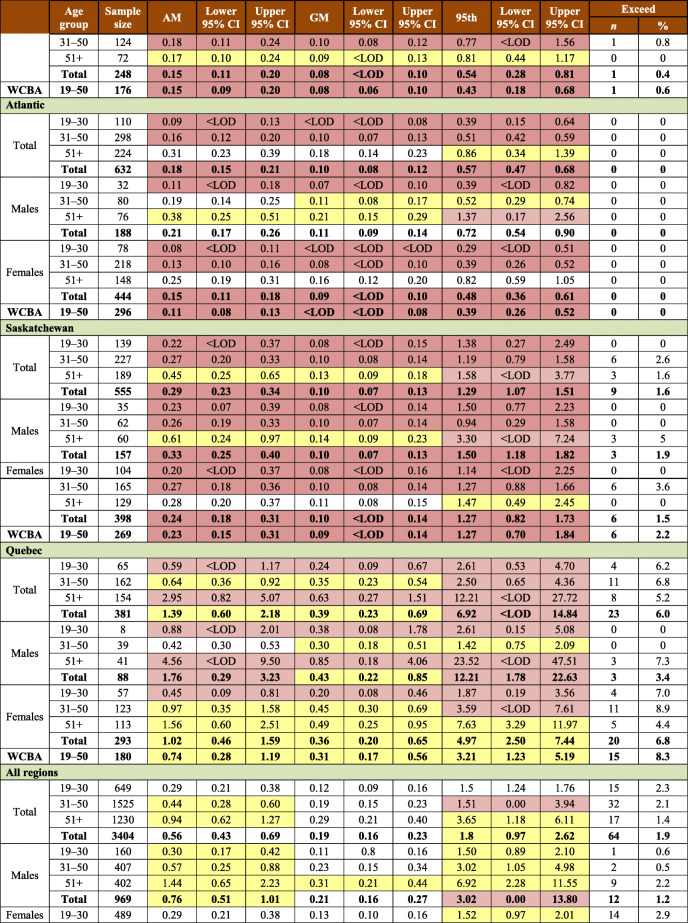

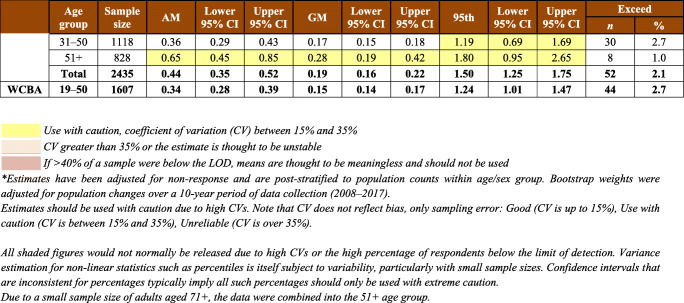
Arithmetic mean (AM), geometric mean (GM), 95th percentile, and exceedances of total mercury in hair concentration (μg/g or ppm) for First Nations adults living on reserve, by region*

### Comparison with the results of the *Methylmercury in Canada *Program

The comparison of mercury exposure of First Nations who participated in the FNFNES (2008–2018) with the historical mercury biomonitoring data in the Canadian First Nations population (1970–1996) (Wheatley & Paradis, 1995; Health Canada, 1999) is presented in Fig. 4 (a-c). The *Methylmercury in Canada* Program (1970–1996) resulted in mercury testing for 38,041 individuals in 529 First Nations (2970 individuals in BC, 801 in AB, 1790 in SK, 9835 in MB, 12,431 in ON, 9514 in QC, and 700 in AT), which demonstrated very high levels of exposure to mercury (the highest level observed was 660 μg/L in Ontario) among the First Nations population who ate fish more frequently, a seasonal cycle of exposure and a steady decrease in mean mercury levels in decades post the 1970s (Health Canada, 1999).

The Health Canada data showed that in the period of time up to the end of 1978, 2.54% of results were at or above 100 μg/L (blood) range. Between 1983 and 1996, this percentage fell to 0.3%. Compared with the historical records, hair Hg concentrations reported in FNFNES are generally lower. In fact, there was not a single FNFNES hair sample that tested at the level equal to or above 30 μg/g of total mercury in hair, which would have required clinical (public health and medical toxicology specialists’) follow-up (Legrand et al., 2010).

The percentage of First Nations who had hair Hg concentrations above the established Health Canada acceptable level (20 μg/L or 6 μg/g) dropped by 20% (from 21.4% to 1.4%) when combining results across all regions (Fig. 4 a-b). Notably, Fig. 4b illustrates the levels of mercury among FNFNES participants 51 years and older, who were young adults in the late 1980s.

Only 0.7% of the entire sample tested in the range equivalent to 20–100 μg/L mercury in blood. The highest mercury result obtained in FNFNES was 23.5 μg/g in hair (Table 1).

To further highlight the differences, applying the new biomonitoring guideline for WBCA to the entire set of FNFNES mercury results (Fig. 4c) shows that 95.5% of the participants had levels of mercury below 2 μg/g, which highlights the threefold decrease in our frame of reference regarding mercury exposure of First Nations people over the last 20 years. Nevertheless, there are still exceedances of the acceptable level guidelines for the general First Nations population and WCBA, as outlined above.

### Comparison with the general Canadian population (CHMS)

The comparison of the FNFNES results on mercury biomonitoring with general population results derived from various phases of the Canadian Health Measures Survey (CHMS) (Health Canada, 2019; Pollock & St-Amand, National Biomonitoring Section, Healthy Environments and Consumer Safety Branch, Health Canada; personal communication, 2019) is illustrated in Table 3. The mean hair Hg concentration among First Nations was higher than that of the general Canadian population in British Columbia and Quebec, but was lower in the Atlantic and Alberta Regions, and was similar in Manitoba, Ontario, and Saskatchewan.

**Table 3:** Comparison of estimates on whole blood total mercury concentrations* (μg/L) of the First Nations populations living on-reserve south of the 60^th^ parallel (FNFNES, 2008–2016) and the Canadian population (Canadian Health Measures Survey (CHMS) cycle 1 (2007–2009), cycle 2 (2009–2011), cycle 3 (2012–2013), and cycle 4 (2014–2015)) aged 19–79 years by sex

Population	Sex	Count (*n*)	%<LOD^a^	AM (95% CI)	GM (95% CI)	10th (95% CI)	25th (95% CI)	50th (95% CI)	75th (95% CI)	90th (95% CI)	95th (95% CI)
FNFNES, BC (2008/09)	Total	487	5.1	2.37 (1.44–3.3)	1.46 (0.99–2.14)	0.24 (0.13–0.35)	0.56 (0.41–0.71)	1.37 (0.6–2.13)	2.98 (1.05–4.92)	6.00 (3.2–8.79)	8.08 (5.48–10.68)
	Female	346	5.5	2.16 (1.5–2.81)	1.39 (1.01–1.91)	0.28 (0.15–0.41)	0.56 (0.38–0.74)	1.29 (0.86–1.73)	2.88 (1.3–4.46)	5.19 (3.8–6.58)	6.16 (5.09–7.23)
	Male	141	4.2	2.57 (1.16–3.98)	1.52 (0.86–2.69)	0.24 (0.04–0.44)	0.56 (0.28–0.84)	1.51 (0.13–2.88)	3.30 (0.65–5.94)	7.82 (2.32–13.31)	8.24 (3.52–12.95)
CHMS cycle 1 (2007–2009)	Total	3567	5.8	1.6 (1.1–2.0)	0.82 (0.66–1.0)	0.17 (0.13–0.21)	0.42 (0.33–0.50)	0.92 (0.73–1.1)	1.8 (1.3–2.3)	3.3^E^ (1.8–4.7)	5.2^E^ (2.4–8.1)
	Female	1888	5.7	1.5 (1.0–1.9)	0.82 (0.64–1.1)	0.18 (0.13–0.23)	0.41 (0.30–0.52)	0.93 (0.71–1.1)	1.8 (1.2–2.3)	3.2^E^ (1.9–4.5)	4.9^E^ (1.9–8.0)
	Male	1679	5.9	1.7 (1.1–2.2)	0.82 (0.67–1.0)	0.16 (0.12–0.20)	0.43 (0.34–0.51)	0.90 (0.74–1.1)	1.8 (1.3–2.4)	3.3^E^ (1.8–4.9)	5.4^E^ (3.0–7.9)
FNFNES, MB (2010)	Total	236	28.4	1.32 (0.34–2.29)	0.53 (0.31–0.9)			0.56 (0.26–0.87)	1.30 (0.41–2.18)	2.68 (–0.08–6.59)	6.27 (0.51–12.02)
	Female	198	28.3	0.86 (0.6–1.13)	0.45 (0.33–0.63)			0.51 (0.26–0.76)	0.85 (0.57–1.14)	2.14 (1.37–2.9)	2.93 (1.45–4.41)
	Male	38	28.9	1.75F (–0.01–3.57)	0.61F (0.27–1.38)			0.57 (0.1–1.04)	1.23 (–0.28–2.74)	4.18 (–0.02–8.59)	6.40 (–0.1–14.37)
FNFNES, ON (2011/12)	Total	744	13.3	1.62E (1.03–2.22)	0.75 0.63–0.9)	0.14F (0.13–0.15)	0.35F (0.29–0.41)	0.67F (0.51–0.84)	1.75F (1.32–2.19)	3.42F (1.74–5.1)	5.39F (1.98–8.81)
	Female	508	14.4	1.35 (1.01–1.7)	0.67 (0.57–0.79)	0.14E (0.14–0.14)	0.32E (0.15–0.49)	0.62E (0.51–0.74)	1.46E (0.93–1.99)	3.22E (2.4–4.03)	4.60E (3.24–5.96)
	Male	236	11.0	1.89E (0.96–2.83)	0.85E (0.61–1.17)	0.14F (–0.01–0.37)	0.38F (0.27–0.5)	0.80F (0.44–1.17)	1.86F (0.97–2.75)	4.00F (1.25–6.75)	6.95F (1.91–11.99)
CHMS cycle 2 (2009–2011)	Total	3706	7.4	1.80 (1.3–2.3)	0.86 (0.68–1.1)	0.16^E^ (<LOD–0.23)	0.29 (0.29–0.51)	0.94 (0.72–1.2)	2.0 (1.6–2.4)	4.0 (2.7–5.3)	6.4^E^ (3.9–9.0)
	Female	1988	7.7	1.60 (1.2–2.1)	0.8 (0.64–1.0)	0.18^E^ (0.10–0.26)	0.40 (0.29–0.51)	0.88 (0.69–1.1)	1.8 (1.3–2.3)	3.4 (2.3–4.5)	5.4^E^ (2.5–8.3)
	Male	1718	7.0	2.00 (1.4–2.7)	0.92(0.7–1.2)	0.16^E^ (<LOD–0.24)	0.42 (0.30–0.55)	1.0 (0.75–1.3)	2.2 (1.6–2.8)	4.2^E^ (2.4–6.0)	7.6^E^ (3.2–12)
FNFNES, AB (2013)	Total	369	40.7	0.74 (0.41–1.08)	0.33 (<LOD–0.42)	<LOD	<LOD	<LODF (<LOD–0.37)	0.70E (0.42–0.99)	1.35F (<LOD–2.65)	3.07E (1.41–4.72)
	Female	248	47.2	0.55 (0.41–0.69)	0.29 (<LOD–0.34)	<LOD	<LOD	<LOD	0.52E (<LOD–0.78)	1.20E (0.83–1.56)	1.91E (0.82–3)
	Male	121	27.3	0.94F (0.29–1.59)	0.38E (<LOD–0.59)	<LOD	<LOD	<LODF (<LOD–0.62)	0.80E (<LOD–1.33)	2.16F (<LOD–4.28)	4.18F (0.81–7.54)
CHMS cycle 3 (2012–2013)	Total	3249	24.1	1.6 (1.1–2.1)	0.91 (0.73–1.1)	<LOD	0.44 (<LOD–0.60)	0.92 (0.71–1.1)	1.8 (1.2–2.3)	3.8^E^ (1.9–5.7)	6.0^E^ (2.8–9.2)
	Female	1642	24.6	1.6 (1.1–2.2)	0.93 (0.77–1.1)	<LOD	0.46^E^ (<LOD–0.64)	0.95 (0.77–1.1)	1.8 (1.3–2.3)	3.8^E^ (1.4–6.3)	F
	Male	1607	23.7	1.6 (1.1–2.2)	0.89 (0.68–1.2)	<LOD	0.42 (<LOD–0.57)	0.90 (0.64–1.2)	1.7^E^ (0.77–2.6)	3.8^E^ (2.0–5.7)	5.9^E^ (2.6–9.2)
FNFNES, AT (2014)	Total	632	41.0	0.72 (0.58–0.85)	0.39 (0.32–0.48)	<LOD	<LOD	0.38E (<LOD–0.56)	0.87 (0.64–1.1)	1.65E (1.3–2.00)	2.31E (1.89–2.73)
	Female	444	46.4	0.58 (0.45–0.72)	0.34 (<LOD–0.42)	<LOD	<LOD	<LOD	0.76E (0.51–1.00)	1.36 (0.96–1.76)	1.94 (1.43–2.45)
	Male	188	28.2	0.85 (0.67–1.03)	0.45 (0.35–0.58)	<LOD	<LOD	0.48E (0.29–0.68)	1.03 (0.76–1.30)	1.90 (1.62–2.19)	2.89 (2.17–3.61)
FNFNES, SK (2015)	Total	555	43.4	1.20 (0.95–1.45)	0.39 (0.28–0.54)	<LOD	<LOD	<LOD (<LOD–0.36)	0.94 (0.29–1.59)	3.42 (2.09–4.75)	5.32 (4.38–6.26)
	Female	398	42.7	1.10 (0.73–1.46)	0.39 (<LOD–0.57)	<LOD	<LOD	<LOD (<LOD–0.42)	0.88 (0.34–1.42)	3.18 (1.43–4.93)	5.08 (3.42–6.75)
	Male	157	45.2	1.30 (0.99–1.61)	0.39 (0.28–0.54)	<LOD	<LOD	<LOD (<LOD–0.34)	1.10 (<LOD–1.94)	3.61 (2.61–4.60)	5.99 (4.69–7.29)
CHMS cycle 4 (2014–2015)	Total	3224	32.1	1.20 (0.98–1.5)	0.7 (0.6–0.82)	<LOD	<LOD	0.72 (0.57–0.88)	1.5 (1.2–1.7)	3.0 (2.2–3.8)	3.8 (2.8–4.8)
	Female	1628	32.5	1.10 (0.89–1.4)	0.68 (0.57–0.81)	<LOD	<LOD	0.72 (0.55–0.90)	1.4 (1.2–1.7)	2.4 (1.7–3.2)	3.6 (3.0–4.3)
	Male	1596	31.6	1.30 (1.1–1.6)	0.72 (0.63–0.84)	<LOD	<LOD	0.76 (0.62–0.91)	1.6 (1.3–1.9)	3.2 (2.4–4.0)	4.2 (3.0–5.4)
FNFNES, QC (2016)	Total	381	22.6	5.80E (2.43–9.17)	1.66E (0.89–3.1)	<LODF (<LOD–0.42)	0.68F (<LOD–1.38)	1.56E (0.83–2.3)	3.86F (0.75–6.97)	13.53F (<LOD–28.82)	27.68F(<LOD–58.34)
	Female	293	22.2	4.43E (1.77–7.09)	1.58F (0.79–3.16)	<LOD (<LOD–0.45)	0.60F (<LOD–1.2)	1.61F (<LOD–3.08)	4.24F (<LOD–9.95)	12.84E (4.62–21.06)	19.88E (10.76–29.00)
	Male	88	23.9	7.21F (1.42–13.00)	1.75E (0.90–3.42)	<LOD (<LOD–0.5)	0.68F (<LOD–1.47)	1.56E (0.95–2.17)	3.06F (<LOD–6.64)	27.68F (<LOD–62.99)	48.83F (7.21–90.45)

*A hair/blood ratio of 250/1 was used to convert hair mercury values to blood mercury concentrations for the FNFNES participants. The equation is as follows: hair value (mg/kg) = (blood value (μg/L) × 250/1000) (Legrand et al., 2010)

*CHMS notes*:

The limits of detection (LOD) for the analytical method are 0.1, 0.1, 0.42, and 0.42 for cycles 1, 2, 3, and 4, respectively

E Use data with caution, CV was between 16.6% and 33.3%

F Data are too unreliable to be published; CV was greater than 33.3%

*FNFNES notes*:

The limit of detection for total mercury in hair was 0.06 ppm (or μg/g)

E Use data with caution; CV was between 15% and 35%

F Estimates are thought to be unstable; CV was greater than 35%

“.” means that the survey estimates could not be calculated

### Comparison with Health Canada guidelines

Overall, there were 64 exceedances of Health Canada’s mercury biomonitoring guidelines (44 WCBA, 8 women aged 51+, 3 men aged 19–50, and 9 men aged 51+). An exceedance was reported if at least one of the three hair segments sampled was above the guidelines. At the regional level, the highest number of participants with hair mercury concentrations exceeding Health Canada’s mercury biomonitoring guidelines was in Quebec (*n* = 23), which represented 6.0% of the total sample and 8.3% of WCBA. In Ontario, a total of 18 respondents (2.4%), with 10 WCBA (3.3%), exceeded the hair mercury guidelines, while in Manitoba, 9 WCBA (4.5%) exceeded the hair mercury guideline of 2 μg/g (Table 2). The distribution of mercury in hair at the 95th percentile indicates that overall, mercury body burden is below the established Health Canada mercury guidelines of 6 μg/g in hair (ranging from 0.16 to 3.6 μg/g across age and sex groups) in all regions except in Quebec. In the Quebec region, the weighted estimate at the 95th percentile for the total First Nations population was 6.92 μg/g, exceeding Health Canada’s guideline of 6 μg/g. It is important to note that the distribution is negatively skewed as 19.4% of tested hair samples were below the LOD. Therefore, it is likely that the 95th percentile hair Hg is underestimating the true level of the top 5% of the population. For WCBA in the Quebec region, the hair mercury concentration at the 95th percentile was 3.21 μg/g, which exceeded the biomonitoring guideline of 2 μg/g.

**Fig. 2:**
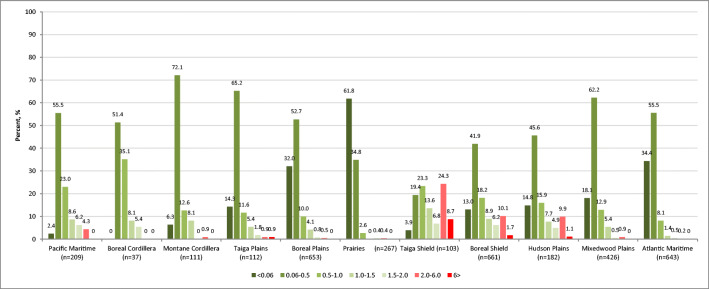
Mercury concentration in hair of participants, by ecozone (percent, %). <2 μg/g in hair—no risk for women of childbearing age (WCBA); 2–6 μg/g in hair—increased risk for WCBA; >6 μg/g in hair—increased risk

**Fig. 3:**
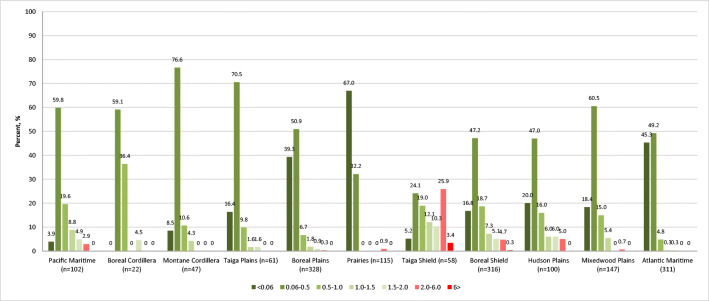
Mercury concentration in hair of WCBA, by ecozone (percent, %). <2 μg/g in hair—no risk for WCBA; 2–6 μg/g in hair—increased risk for WCBA; >6 μg/g in hair—increased risk

**Fig. 4:**
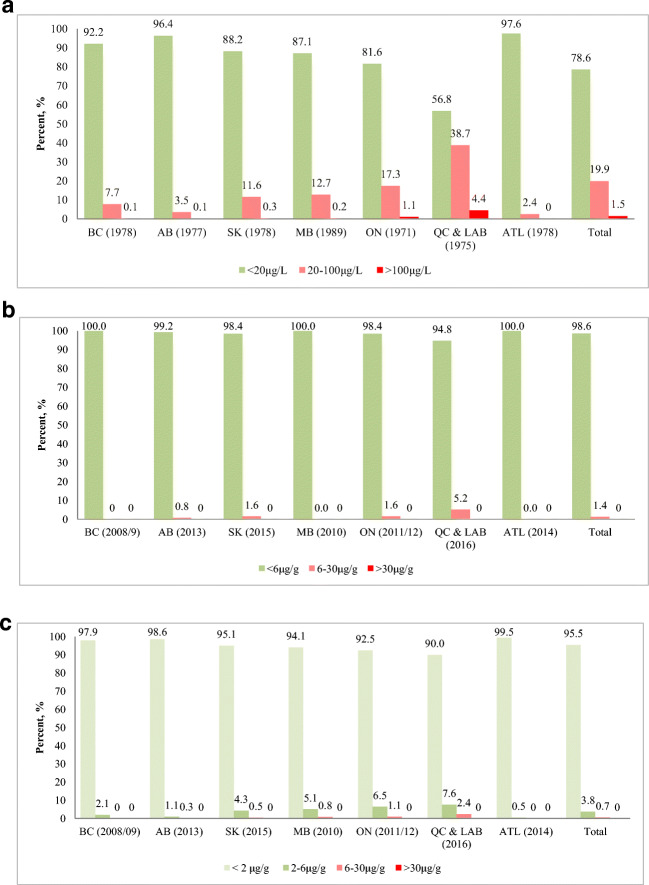
Comparison of mercury exposure in the FNFNES First Nations participants (2008–2016) with the historical levels of methylmercury exposure in First Nations in Canada (1970–1996). **a** Blood methylmercury concentrations in First Nations in Canada, by region (1970–1996) (Health Canada, 1999). *<*20 μg/L in blood—acceptable; 20–100 μg/L in blood—increased risk; >100 μg/L in blood—at risk. **b** Hair mercury concentrations in First Nations aged 51 years and older, by region, FNFNES (2008–2016). <6 μg/g in hair—acceptable; 6–30 μg/g in hair—increased risk; >30 μg/g in hair—at risk*.* **c** Hair mercury concentrations in First Nations (total population) by region, FNFNES (2008–2016). <2 μg/g in hair—no risk for WCBA; 2–6 μg/g in hair—increased risk for WCBA; 6–30 μg/g in hair—increased risk; >30 μg/g in hair—at risk

The analysis by ecozone demonstrated significant differences in the profiles of mercury exposure among the study participants (Figs. 2 and 3). The northern ecozones are characterized by a higher frequency of elevated Hg exposures. Of the 23 exceedances of Health Canada’s biomonitoring guideline for the general population (6 μg/g), 22 were in the northern ecozones, namely the Taiga Shield (*n* = 9), Boreal Shield (*n* = 11), and Hudson Plains (*n* = 2), which represented 8.7%, 1.7%, and 1.1% of the total population in each ecozone, respectively. Most exceedances were among participants aged 51 and older (Fig. 4). Most of the 44 exceedances among WCBA (2 μg/g guideline) were found in the Taiga Shield (*n* = 17 or 29.3%) followed by Boreal Shield (*n* = 16 or 5.0%), Hudson Plains (*n* = 5 or 5.0%), and Pacific Maritime (*n* = 3 or 2.9%).

## Discussion

The FNFNES mercury biomonitoring results, especially for the British Columbia region, appear similar to the First Nations Biomonitoring Initiative (AFN, 2013), which was led by the Assembly of First Nations, in collaboration with FNIHB, and developed the first national estimate of the body burden of 97 environmental chemicals, including total mercury (Table 4). The examination of percentiles, while similar to FNFNES, is made unreliable due to the high coefficient of variation. Our results showed a clear geographic variation with a strong south-north increasing gradient of mercury hair levels in all regions except for BC. In the BC region, we found that coastal communities had higher Hg exposure than the inland First Nations, which could be explained by their greater reliance on fish/seafood. But, unlike in BC, QC region coastal communities had lower Hg exposure (e.g., in Atlantic Maritime ecozone) compared with the inland FNs.

**Table 4:** Mercury (total)—Arithmetic means, geometric means, and selected percentiles of whole blood concentrations (μg/L) for on-reserve and crown land populations aged 20 years and older, reproduced from Table 7.1 in the AFN publication, First Nations Biomonitoring Initiative (2011)

Population	Sex	Count (*n*)	% <LOD^a^	AM (95% CI)	GM (95% CI)	10th (95% CI)	25th (95% CI)	50th (95% CI)	75th (95% CI)	90th (95% CI)	95th (95% CI)
FNBI (2011)	Total	473	8.90	2.47E (1.19–3.75)	0.95E (0.51–1.77)	0.12E (0.04–0.19)	F	F	F	6.37E (3.45–9.29)	9.28E (5.36–13.20)
	Female	285	9.80	2.46E (1.09–3.84)	F	.	F	F	F	6.42E (2.60–10.25)	9.85E (5.40–14.30)
	Male	188	7.40	2.47E (1.22–3.72)	0.97E (0.55–1.71)	0.13E (0.06–0.20)	F	F	3.79E (1.34–6.24)	6.21E (3.99–8.42)	8.67E (5.25–12.09)

Note: If >40% of samples were below LOD, the percentile distribution is reported but means were not calculated

E means that the survey estimates should be used with caution; CV was between 16.6% and 33.3%

F means that the survey estimates were too unreliable to be published, CV was above 33.3%

“.” means that the survey estimates or their coefficient of variation could not be calculated

At the same time, results reported by region showed that the highest number of participants with hair mercury concentrations exceeding Health Canada’s mercury biomonitoring guidelines were in Quebec. These results agree with the dietary Hg exposure results reported by Chan et al. (2021a) in this issue. We had previously examined the relationship between the estimated mercury intake from traditional foods (Chan et al., 2021a) and hair mercury concentrations of the participants. While we found a significant positive correlation, only 9% of the variance of hair mercury could be explained by the estimated mercury intake from traditional food (Chan et al., 2019b). These results suggest that even though both the dietary estimate and biomonitoring showed a similar trend of exposure, there were significant variations. While exposure biomarkers such as hair Hg concentrations are established tools for monitoring and estimating Hg exposure in human populations, it has been widely reported that discrepancies exist in the relationships between actual exposure as measured through dietary intake and hair Hg concentrations (Canuel et al., 2006). Known causes for the variations include drivers such as genetic and epigenetic factors of the individuals, bioavailability factors such as cooking methods and presence of certain nutrients, and the individual gut microbiome affecting the bioavailability of Hg (Eagles-Smith et al., 2018). There are also intrinsic inaccuracies associated with both the dietary estimates and biomonitoring results and the possibility of other sources of mercury exposure such as dental amalgams (Clarkson et al., 2003). Nevertheless, our results can be used to guide risk communication efforts and public health education. For example, mercury risk communication can be focused more on the First Nations in northern ecozones and, particularly, in Quebec.

A key finding of this study is the high number of WCBA whose hair Hg concentration was below the LOD. Specifically, 33.3% of samples for WCBA in Manitoba, 46% in Alberta, 45.6% in Atlantic, and 37.2% in Saskatchewan regions were below the LOD for mercury. These results suggest that one third to half of the WCBA in these regions only consumed fish that had very low Hg concentrations or were consuming very small amounts of fish. This may be important because, as the joint FAO/WHO expert consultation on the risks and benefits of fish consumption pointed out in 2010 (FAO/WHO, 2011), the consumption of fish provides energy, protein, and a range of other important nutrients, including the long-chain *n*-3 polyunsaturated fatty acids (LCn3PUFAs). When comparing the benefits of LCn3PUFAs with the risks of methylmercury among WCBA, the expert group concluded that maternal fish consumption lowered the risk of suboptimal neurodevelopment in their children, compared with children of women who did not consume fish in most circumstances evaluated. Specifically, with an upper estimate of methylmercury risk, the neurodevelopmental risks of not consuming fish regularly exceed the risks of eating fish up to seven 100 g servings per week for all fish containing less than 0.5 μg/g methylmercury and up to two servings per week for fish with greater than 8 mg/g EPA plus DHA and up to 1 μg/g methylmercury (FAO/WHO, 2011). With this in mind, the results of FNFNES may point to the need for the promotion of fish consumption among First Nations WCBA in Manitoba, Saskatchewan, Alberta, and Atlantic regions.

The comparison between the FNFNES and CHMS suggests that, in general, First Nations exposures to mercury are similar to low background levels of exposure found in the Canadian general population. However, there are still relatively high levels of exposure to mercury in subgroups (95th percentile) of the First Nations population (BC and QC). In Quebec, the mercury body burden of First Nations women at the 95th percentile was 5 times higher than the 95th percentile in the general Canadian population. Therefore, these findings suggest that the balance between the need to ensure consumption of fish and to decrease mercury exposure should be focussed primarily on decreasing mercury exposure among Quebec First Nations women, by promoting the consumption of smaller fish and non-piscivorous fish species (e.g., white fish) only.

Our studies have a number of limitations. There is an under-representation of males in the sample (Table 1). This was explained by the unavailability of males at the time of the survey and sampling, the high prevalence of very short haircuts among males that did not allow the application of the FNFNES sampling protocol, and the lack of interest in sampling among male community members. There may have been cultural barriers that prevented some people from contributing hair for sampling due to cultural beliefs that this would not be appropriate. This limitation of the sampling, despite statistical adjustments, may have implications on the representativeness of these results for the First Nations males.

Some comparisons made in this study are merely descriptive observations that do not have rigorous statistical techniques applied in support of the comparison. There remains a gap in the design of national studies that would be able to apply the same methodology to Indigenous and non-Indigenous participants within the same time frame.

Although a high coefficient of variation (CV) is expected when conducting a national-level sampling among First Nations, due to the inherent diversity of cultures and lifestyles present among First Nations, it was surprising to observe it to this extent in the regionally representative stratified random sampling. This may indicate the presence of lifestyle diversity within individual First Nations, which should be further examined as a part of continued FNFNES data analysis and as an objective for future research.

If more than 40% of the sample is below the LOD, which was observed in several age and sex groups, the means are biased and should not be used. Furthermore, results should be used with caution in the case where the CV is between 15% and 35%; and estimates are considered unreliable if the CV is greater than 35% (Table 2).

The approach used by this study was to assess the risk of mercury exposure at the population level. There may be individuals who might be more sensitive to mercury, and their concerns, if any, will need to be addressed by the local physicians.

## Conclusion

In general, the FNFNES results suggest that current mercury exposure no longer presents a significant clinical health risk in most of the First Nations population south of the 60^th^ parallel across Canada. Nevertheless, the extent of exposures that was found at the 95th percentile, particularly among First Nations in Quebec, may continue exerting subtle subclinical health impacts. Therefore, mercury exposure continues to be an ongoing environmental public health concern that requires continued monitoring and assessment. Specifically, women of childbearing age and older individuals (51 years and over) living in northern ecozones and Quebec tend to have higher mercury exposure that often exceeds Health Canada’s guidelines. Further studies in these areas need to investigate the prevalence of higher mercury exposures and to provide coherent risk management advice on the importance of fish consumption and the reduction of exposure to mercury. Additional research is needed to better assess mercury exposure among First Nations men. Continued mercury biomonitoring is essential, particularly due to the importance of fish in the traditional diet of First Nations in Canada (Marushka et al., 2021).
